# Type D personality is a predictor of prolonged acute brain dysfunction (delirium/coma) after cardiovascular surgery

**DOI:** 10.1186/s40359-019-0303-2

**Published:** 2019-05-02

**Authors:** Yujiro Matsuishi, Nobutake Shimojo, Takeshi Unoki, Hideaki Sakuramoto, Chiho Tokunaga, Yasuyo Yoshino, Haruhiko Hoshino, Akira Ouchi, Satoru Kawano, Hiroaki Sakamoto, Yuji Hiramatsu, Yoshiaki Inoue

**Affiliations:** 1 Department of Emergency and Critical Care Medicine, Faculty of Medicine, University of Tsukuba, Tsukuba, Ibaraki Japan; 2 Department of Adult Health Nursing, School of Nursing, Sapporo City University, Sapporo, Japan; 3 Adult Health Nursing, College of Nursing, Ibaraki Christian University, Hitachi, Ibaraki Japan; 4 Department of Cardiovascular Surgery, Faculty of Medicine, University of Tsukuba, Tsukuba, Ibaraki Japan; 5 Department of Nursing, Kanto Gakuin University College of Nursing, Yokohama, Kanagawa Japan

**Keywords:** Delirium, Delirium/coma days, Type D personality, Depression, Thoracic surgery, Intensive care units, Critical care

## Abstract

**Background:**

Previous studies have shown a relationship between delirium and depressive symptoms after cardiac surgery with distress personalities linking to negative surgical outcomes. The aim of the present study is to further investigate the association between patients with Type D (distressed) personality with regards to delirium after cardiac surgery.

**Methods:**

We conducted a consecutive-sample observational cohort pilot study with an estimated 142 patients needed. Enrollment criteria included patients aged ≥18 years who were undergoing planned cardiovascular, thoracic and abdominal artery surgery between October 2015 to August 2016 at the University of Tsukuba Hospital, Japan. All patients were screened by Type-D Personality Scale-14 (DS14) as well as the Hospital Anxiety and Depression Scale (HADS) the day before surgery. Following surgery, daily data was collected during recovery and included severity of organ dysfunction, sedative/analgesic exposure and other relevant information. We then evaluated the association between Type D personality and delirium/coma days (DCDs) during the 7-day study period. We applied regression and mediation modeling for this study.

**Results:**

A total of 142 patients were enrolled in the present study and the total prevalence of delirium was found to be 34% and 26% of the patients were Type D. Non-Type D personality patients experienced an average of 1.3 DCDs during the week after surgery while Type D patients experienced 2.1 days over the week after surgery. Multivariate analysis showed that Type D personality was significantly associated with increased DCDs (OR:2.8, 95%CI:1.3–6.1) after adjustment for depressive symptoms and clinical variables. Additionally, there was a significant Type D x depression interaction effect (OR:1.7, 95% CI:1.2–2.2), and depressive symptoms were associated with DCDs in Type D patients, but not in non-Type D patients. Mediation modeling showed that depressive symptoms partially mediated the association of Type D personality with DCDs (Aroian test =0.04).

**Conclusions:**

Type D personality is a prognostic predictor for prolonged acute brain dysfunction (delirium/coma) in cardiovascular patients independent from depressive symptoms and Type D personality-associated depressive symptoms increase the magnitude of acute brain dysfunction.

## Background

Delirium is a common post-surgical neuropsychological complication among cardiac patients and onset occurs rapidly due to the development of physiological abnormalities characterized by fluctuating course, attention deficits, disorganized thinking, and an altered level of consciousness [1].The prevalence of delirium within this post-surgical, cardiac patient population is reported to be between 26 to 52% [2]. This figure is in line with previous studies which report that preoperative cognitive impairment and depression in cardiac surgical patients are associated with greater risk of developing delirium [3, 4]. In addition, risk of delirium increases cumulatively with intraoperative and postoperative factors, such as longer cardiopulmonary bypass times [5] and/or use of benzodiazepine [6]. Importantly, delirium was independently associated with negative outcomes, such as higher mortality [7], decline in cognitive ability [8], increased length of stay and hospital readmissions [8]. However, outside of the prevalence, duration of delirium dose affect mortality [9]. Additionally, reports have measured delirium associated with terminal conditions [10] and from this insight, the concept of measuring both delirium and coma days was born [11–13]. The main concept is that psychiatric disorders can often manifest alongside physical ailments and even if the physical condition causes the initial psychiatric insult, ongoing depressive symptoms can enact a positive feedback loop to worsen the physical condition. To this end, previous studies reported that depressive symptoms are associated with delirium in cardiac patients [14]. However, a recent study reported that heart disease outcomes are not based on psychiatric condition alone but also patient personalities [15–19]. The distress personality (also known as Type D) is based on personality type and is defined by complex and highly negative emotions plus social inhibition [20] This total personality is associated with increase depressive symptoms [21]. Surprisingly, about 30% of cardiac surgery patients that carry this personality [22] suffer adverse consequences [23] and previous research showed a significant association between Type D personality and hard endpoint-adjusted hazard ratios (HR:2.24, 95% CI [1.37–3.66]) in meta-analysis of 12 studies on 5341 patients [24]. Despite this initial evidence linking Type D personality with hazard ratios, a full explanation of the correlation between personality and postoperative delirium which lead to high mortality is still lacking. While previous research has reported that personality traits of neuroticism and conscientiousness are associated with delirium in hip fracture patients [25] another report found no association between Type D personality and delirium [26]. There is still a lack of associative evidence for Type D personality, delirium and the mediating effects of depressive symptoms for this relationship. Some points of improvement were noted in this previous study allowing for closer examination into important factors such as the severity and duration of delirium/coma take account for better patient outcomes.

We hypothesize that a Type D personality affects postoperative delirium/coma days and by using regression and mediation modeling, the present study was able to revisit the association between Type D character and the development of postoperative delirium/coma days after cardiac surgery.

## Material and methods

### Patient selection

A list of enrolled and approved patients was obtained by operation room staff a week before surgery and enrollment criteria included patients aged ≥18 years that were undergoing scheduled cardiovascular, thoracic and abdominal artery operations between October 2015 and August 2016. Patients were excluded if they had stroke, were deaf or otherwise unable to speak, or had current or previous major depression. This information was obtained from medical records. The Institutional Review Board (IRB) of the University of Tsukuba Affiliated Hospital approved the present study (H27–085) and written informed consent was obtained from patients prior to surgery.

### Data collection prior to surgery

We recorded baseline preoperative factors, including age, sex, baseline medical history, and cardiac function, and calculated the European System score for Cardiac Operative Risk EvaluationII (EuroSCOREII) from these data [27] . EuroSCOREII is a cardiac risk score for predicting mortality after cardiac surgery that takes into account patient-related factors, cardiac-related factors, previous cardiac surgery, and operation-related factors. The validation of the EuroSCOREII with Japanese patients has been previously reported [27]. All patients underwent the following evaluations the day before the surgery: (a) the Type-D personality Scale-14 (DS14) [28]; (b) the Hospital Anxiety and Depression Scale (HADS) [29] and (c) the Mini-Mental State Examination (MMSE) [30]. The DS14 was specifically developed to assess Negative Affectivity (NA) and Social Inhibition (SI). This scale contains fourteen items and these subscales consist of seven items, and each item is rated from false (0) to true (4) on a 5-point Likert scale. Scores equal to or above 10 on both NA and SI were used to determine a Type D personality. HADS is a self-administered scale for the evaluation of anxiety and depression in non-psychiatric patients. Each item is rated on a 4-point Likert scale and increases measure degree of severity. In the present study, only the depressive HADS scale was used. The MMSE was used to assess presence and severity of cognitive impairment. The validation of the Japanese versions of DS14, HADS and MMSE has been previously reported [31–34]. DS14 and HADS were provided by paper and scoring was done after the experimental period, blinding the researchers to patient Type D status during testing.

### Intra- and post-operative data collection

Intraoperative data, including aortic clamping time, was recorded. Post-operative daily data, including severity of organ dysfunction calculated by modified Sequential Organ Failure Assessment (mSOFA) and Benzodiazepine, Propofol, Dexmedetomidine dosage, were collected during ICU and general ward stays. Modified Sequential Organ Failure Assessment (mSOFA) is an assessment score calculated with SpO2/FiO2, liver function, cardiovascular, hypotension, central nervous system function, and renal creatinine levels. This system has been validated as a good predictor of post-operative mortality [35].

### Delirium assessment

Delirium and coma were assessed using the Richmond Agitation - Sedation Scale (RASS) [36] and Confusion Assessment Method for the ICU (CAM-ICU) [37] twice daily for the 7-day study period. The assessments were all performed by IRB-approved researchers. Patients with RASS − 4 and − 5 were determined to be comatose and if delirium/coma was observed even once for a given day, it was noted that delirium/coma was prevalent for that particular day.

### Delirium/coma days (DCDs)

DCDs are defined as days acute brain dysfunction (delirium and coma) within the study period. Delirium observation, however, took into account the comatose days to avoid lead time bias. Care was taken when recording both delirium and coma to avoid focusing on one of the DCDs conditions at the exclusion of the other (as seen in previous reports) which could have skewed or biased the data [11, 12].

## Statistical analysis

### Sample size calculations

Before this study, we conducted a month-long pilot study where a total of 22 patients, were enrolled and we observed a mean of 0.7 (SD ± 1.4) delirium/coma days (DCDs) in the Type D personality group and a mean of 0.2 (SD ± 0.3) DCDs in the control group. The sample size was calculated with the software G * Power 3.1. Using Wilcoxon-Mann-Whitney testing, and effect size was d = 0.49 based on the pilot study. We determined that a sample size of 142 patients would be required for a significance level (α) of 0.05 and test power (1-β) of 0.80.

### Regression modeling

The outcomes of interest were DCDs within the 7-day study period. DCDs are defined as days with acute brain dysfunction (delirium and coma) within the study period. Because previous studies have noted a heavily skewed distribution of DCDs, we instead decided to use Proportional Odds Logistic Regression (POLR), which does not require the normal distribution, in examining the relationship between Type D personality and DCDs. Furthermore, we also adjusted for the following additional covariates chosen a priori in our model: EuroSCOREII, mSOFA without a central nervous system component, use of sedative medicine, and MMSE. EuroSCOREII for adjusting patient baseline characteristics including sex, age, history of complications, and intraoperative factors including urgency and intervention procedures. We used mSOFA for adjusting for daily severity of the patient. As central nervous system (CNS) components would be correlated with the outcome of interest we excluded this component to protect the integrity of our analysis. Additionally, The variance inflation factor (VIF) were observed to assess multicollinearity among the variables. As previous studies reported [38, 39], we tested continuous values of SI and NA (which are components of Type D personality) independently as a sub-analysis.

### Interaction

As Type D personality and depressive symptoms are generally considered co-morbid, and previous studies reported that having these two factors suspected to inflate bad outcomes for cardiac patients [40, 41]. Therefore, we attempted to construct an interaction model. Interaction modeling can analyze the relationship of the inflation between two factors (covariates) for outcome of the interest. Although the basic assumption of regression modeling is the independence of each factor, we suspected a significant interaction and therefore used a two-step process where we first constructed an isolated main effect model (model 1) then iteratively included interaction modeling (model 2). In model 2, the odds ratio of the main effect (Type D personality and depressive symptoms) was not significant, possibly due to the ability to capture only a segment of the main effect.

### Mediation modeling

To determine the mediating effect of depressive symptoms on the relationship between Type D personality and DCDs, mediation analyses were conducted using the Baron and Kenny approach [42] (bootstrapping method and Aroian testing) [43] and adjusted for the same covariate factors in regression modeling. All statistical analyses were performed using SPSS version 25 (SPSS, Inc., Chicago, IL).

## Results

### Patient characteristics

**Fig. 1:**
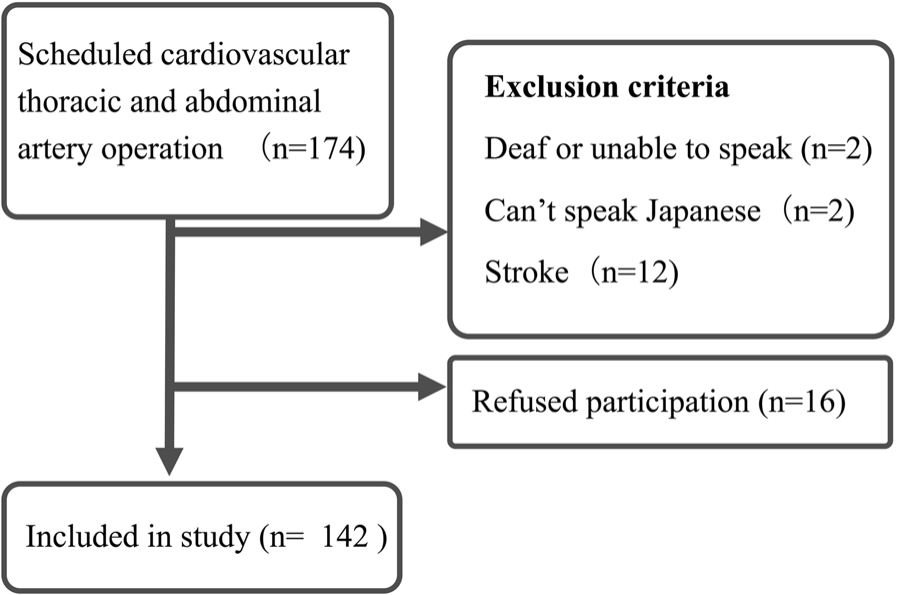
Participant flow chart. This figure shows participant flow chart including exclusion criteria, and final enrollment patients for the investigation

From October 2015 to August 2016, we enrolled a total of 142 patients (see Fig. 1 illustrating participant flow).

Of the 174 patients, the following two groups were excluded from the study: A) 16 patients: 2 deaf or unable to speak, 2 could not speak Japanese and 12 had stroke B) 16 patients that freely exercised their legal right to refuse participation. Table 1 presents baseline patient study characteristics.

**Table 1:** Baseline characteristics of study patients

variable	Total population *N* = 142	Type D personality *N* = 37	Non-Type D personality *N* = 105
Age ± SD	67 ± 14	64 ± 13	67 ± 14
Male n (%)	90 (63)	24(64)	66(62)
Surgical procedure n (%)			
CABG	26 (18)	7 (18)	19 (18)
CABG+Valve surgery	10 (7)	2 (5)	8 (7)
Valve surgery	65 (45)	14 (37)	51 (48)
Thoracic blood vessel replacement	7 (4)	3 (8)	4 (3)
Thoracic blood vessel replacement+VALVE surgery	6 (4)	1 (2)	5 (4)
Abdominal blood vessel replacement	5 (3)	1 (2)	4 (3)
Endovascular aortic repair	16 (11)	5 (13)	11 (10)
ASD/VSD closer	2 (1)	1 (2)	1 (1)
Heart tumor resection	3 (2)	2 (5)	1 (1)
Ventricular aneurysm resection	2 (1)	1 (2)	1 (1)
MMSE ± SD	28 ± 1.52	28 ± 1.50	28 ± 1.53
Depressive symptom ^a^ ± SD	1.9 ± 2.7	3.0 ± 2.9	1.6 ± 2.5
DS 14			
Negative Affectivity (NA)	6.6 ± 6.0	14.8 ± 4.2	3.7 ± 3.2
Social Inhibition (SI)	8.7 ± 6.6	16.0 ± 4.7	6.2 ± 5.1
EuroSCOREII ± SD	2.0 ± 2.0	1.7 ± 1.5	2.1 ± 2.1
Aortic clamping times, min (IQR)	135 (0, 206)	136 (34, 214)	135 (0, 208)
mSOFA ^b^ ± SD	3.5 ± 2.1	3.4 ± 1.9	3.5 ± 2.2
Benzodiazepine (mg/kg/day) ^b^ ± SD	0.06 ± 0.5	0.06 ± 0.41	0.06 ± 0.53
Propofol (mg/kg/day) ^b^ ± SD	2.8 ± 6.6	3.0 ± 7.3	2.3 ± 4.1
Dexmedetomidine (μg/kg/day) ^b^ ± SD	0.8 ± 3.0	0.6 ± 1.2	0.9 ± 3.4
Prevalence of delirium n (%)	49 (34)	17 (45)	32 (30)
Delirium/coma days ± SD	1.5 ± 1.7	2.1 ± 1.9	1.3 ± 1.6
Coma days ± SD	0.9 ± 1.1	0.9 ± 1.0	0.8 ± 1.1
Delirium days ± SD	0.6 ± 1.0	1.1 ± 1.5	0.4 ± 0.8

a: measured by Hospital Anxiety and Depression Scale (HADS)

b: used average of 7 days

*IQR* interquartile range, *SD* standard deviation, *MMSE* mini-mental state examination, *EuroSCOREII* European System for Cardiac Operative Risk Evaluation II, *mSOFA* modified Sequential Organ Failure Assessment

**Fig. 2:**
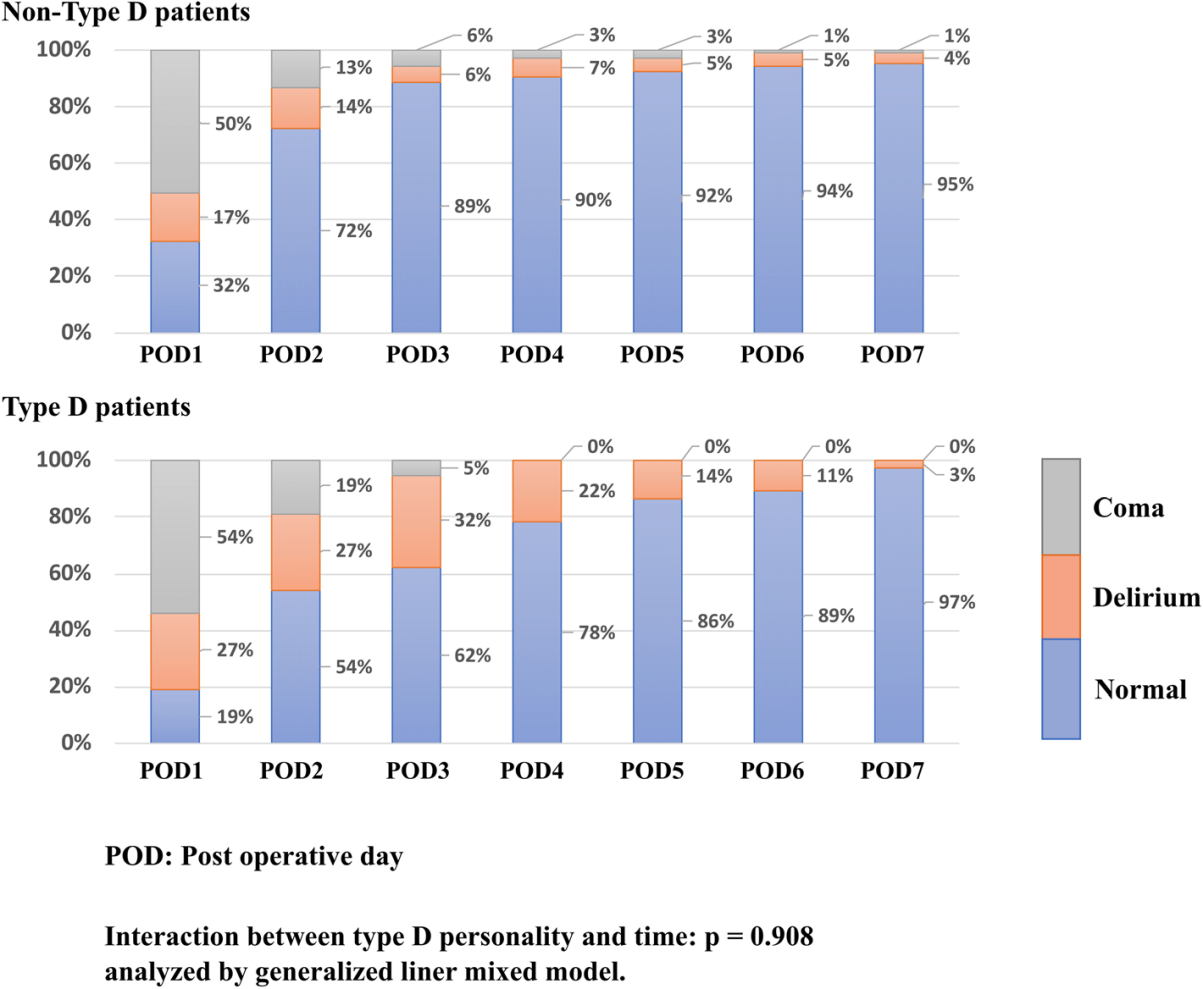
Distribution of normal, delirium, and coma days, stratified by Type D personality. This is the distribution of normal, coma, delirium days for normal and Type D personality

45% of the patient takes valve surgery and the median age at enrollment was 67 (± 14) and 63% of the patients were male. The average EuroSCOREII was 2.0 (± 2.0) and the average of 7-days modified Sequential Organ Failure Assessment was 3.5 (± 2.1). Non-Type D personality patients experienced coma days average of 0.8 ± 1.1 during the week after surgery while Type D patients experienced 0.9 ± 1.0, and Non-Type D personality patients experienced a delirium average of 0.4 ± 0.8 during the week after surgery while Type D patients experienced 1.1 ± 1.5, thus Non-Type D personality patients experienced 1.3 ± 1.6 DCDs during the week after surgery while Type D patients experienced 2.1 ± 1.9 DCDs over the week after surgery (Fig. 2). All patients survived during the study period. Out of the 49 patients (34%) with delirium in total population and 32 patients (30%) in Non-Type D personality 17 patients (45%) in Type D personality patients experienced delirium, 37 patients (26%) were found to have a Type D personality.

### Regression modeling

VIF was less than 3. Therefore, multicollinearity was considered not to be problematic. Type D personality factors [odds ratio (OR) = 2.8, 95% confidence interval (CI) = 1.3–6.1], HADS-Depression (OR = 1.1, 95% CI = 1.0–1.3), mSOFA (OR = 1.7, 95% CI = 1.3–2.2), Benzodiazepine (OR = 9.8, 95% CI = 2.4–40.3) and Propofol (OR = 1.1, 95% CI = 1.0–1.2) were associated with significantly increased DCDs (Table 2). This indicates that these factors were independently associated with prolonged acute brain dysfunction in the 7-day post-operative period. We also tested continuous values of SI and NA (which are components of Type D personality) independently as a sub-analysis NA (OR = 1.09, 95% CI = 1.03–1.15) and SI (OR = 1.05, 95% CI = 1.0–1.1) themselves were also associated with significantly decreased DCDs (Table 3) and NA and SI interaction was not significant. (OR = 0.9, 95% CI = 0.9–1.0) (Table 4).

**Table 2:** Regression model for prolonged delirium/coma days

	Multivariate model 1 OR (95% CI) ^a^	VIF	Multivariate model 2 OR (95% CI) ^a^	VIF
EuroSCOREII	1.1 (0.9–1.3)	1.3	1.1 (0.9–1.3)	1.3
MMSE	0.9 (0.7–1.1)	1.1	0.9 (0.7–1.1)	1.1
Type D personality (Present or not)	2.8 (1.3–6.1)^*^	1.0	2.4 (5.4–1.0)^*^	1.1
Depressive symptoms ^b^	1.1 (1.0–1.3)^*^	1.1	0.9 (0.8–1.1)	1.7
Aortic clamping time	0.9 (0.9–1.0)	1.4	0.9 (0.9–1.0)	1.4
mSOFA ^c^	1.7 (1.3–2.2)^*^	1.7	1.7 (1.3–2.7)^*^	1.7
Benzodiazepine ^d^	9.8 (2.4–40.3)^*^	1.1	16.1 (3.7–69.8)^*^	1.1
Propofol ^d^	1.1(1.0–1.2) ^*^	1.9	1.1(1.1–1.3) ^*^	1.9
Dexmedetomidine ^e^	1.1(0.9–1.2)	1.7	1.1(0.9–1.2)	1.7
Type D personality × Depressive symptom ^f^			1.7 (1.2–2.2)^*^	1.6

a: *P* values obtained from Ordered Logistic Regression *P value<0.05

b: measured by Hospital Anxiety and Depression Scale (HADS)

c: Exclude GCS, used average of 7 days

d: Used average of 7 days. mg/day/kg

e: Used average of 7 days. μg/day/kg

f: Centering was performed

*MMSE* mini-mental state examination, *EuroSCOREII* European System for Cardiac Operative Risk Evaluation II, *mSOFA* modified Sequential Organ Failure Assessment

**Table 3:** Sub-analysis of each tendency of regression model for prolonged delirium/coma days

	Multivariate model 3 OR (95% CI) ^a^	VIF	Multivariate model 4 OR (95% CI)^a^	VIF
EuroSCOREII	1.1 (0.9–1.3)	1.3	1.1 (0.9–1.3)	1.2
MMSE	0.9 (0.7–1.1)	1.0	0.9 (0.7–1.3)	1.1
Negative Affectivity (NA)	1.09 (1.03–1.15)^*^	1.0		
Social Inhibition (SI)			1.05 (1.0–1.1) ^*^	1.0
Depressive symptoms^b^	1.1 (1.0–1.3)^*^	1.1	1.1 (1.0–1.3) ^*^	1.1
Aortic clamping time	0.9 (0.9–1.0)	1.0	0.9 (0.9–1.0)	1.0
mSOFA^c^	1.7 (1.3–2.2)^*^	1.6	1.6 (1.2–2.1)^*^	1.6
Benzodiazepine^d^	9.9 (2.4–40.2)^*^	1.0	9.8 (2.3–40.9)^*^	1.0
Propofol^d^	1.1(1.0–1.2)^*^	1.8	1.1(1.0–1.2) ^*^	1.8
Dexmedetomidine^e^	1.1(0.9–1.2)	1.7	1.1(0.9–1.3)	1.7

a: *P* values obtained from Ordered Logistic Regression *P value<0.05

b: measured by Hospital Anxiety and Depression Scale (HADS)

c: Exclude GCS, used average of 7 days

d: Used average of 7 days. mg/day/kg

e: Used average of 7 days. μg/day/kg

*MMSE* mini-mental state examination, *EuroSCOREII* European System for Cardiac Operative Risk Evaluation II, *mSOFA* modified Sequential Organ Failure Assessment

**Table 4:** Sub-analysis of each tendency’s regression modeling interaction for prolonged delirium/coma days

	Multivariate model 5 OR (95% CI)^a^	VIF
EuroSCOREII	1.1 (0.9–1.3)	1.3
MMSE	0.9 (0.9–1.1)	1.1
Negative Affectivity (NA)^b^	1.0 (1.0–1.1)^*^	2.5
Social Inhibition (SI)^b^	1.0(0.9–1.0)	1.8
Negative Affectivity (NA) × Social Inhibition (SI)	0.9(0.9–1.0)	1.7
Depressive symptoms^c^	1.1 (1.0–1.3)^*^	1.1
Aortic clamping time	0.9 (0.9–1.0)	1.0
mSOFA^d^	1.7 (1.3–2.2)^*^	1.6
Benzodiazepine^e^	11 (2.6–46.2)^*^	1.0
Propofol^e^	1.1(1.0–1.2)^*^	1.8
Dexmedetomidine^f^	1.1(0.9–1.3)	1.7

a: *P* values obtained from Ordered Logistic Regression *P value<0.05

b: Centering was performed

c: measured by Hospital Anxiety and Depression Scale (HADS)

d: Exclude GCS, used average of 7 days

e: Used average of 7 days. mg/day/kg

f: Used average of 7 days. μg/day/kg

*MMSE* mini-mental state examination, *EuroSCOREII* European System for Cardiac Operative Risk Evaluation II, *mSOFA* modified Sequential Organ Failure Assessment

### Moderator model

Model 2 for DCDs included interaction between Type D personality and depressive symptoms, and this interaction was found to be significant (Type D personality×depressive symptoms: OR = 1.7, 95% CI = 1.2–2.2). (Table 2).

**Fig. 3:**
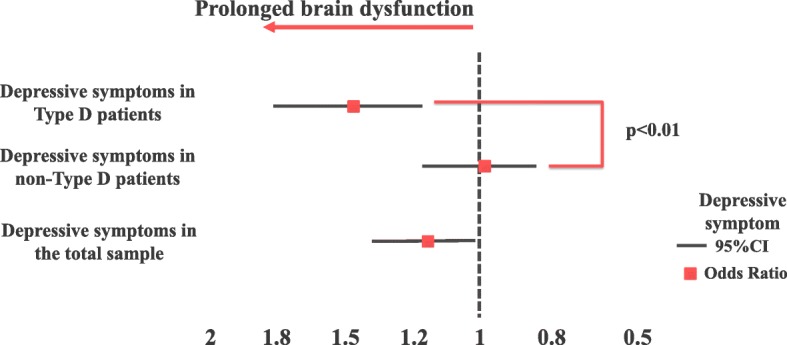
Association of depressive symptoms with prolonged brain dysfunction, stratified by Type D personality. The interactive effect of Type D personality and depressive symptoms on DCDs. Adjusted for the covariate factors used in regression modeling

This interaction effect indicates that Type D personality moderated the association of depressive symptoms with DCDs; i.e., depressive symptoms had a deleterious effect in terms of prolonged brain dysfunction among Type D patients, but depressive symptoms were not associated with DCDs in non-Type D patients (Fig. 3).

### Mediation model

**Fig. 4:**
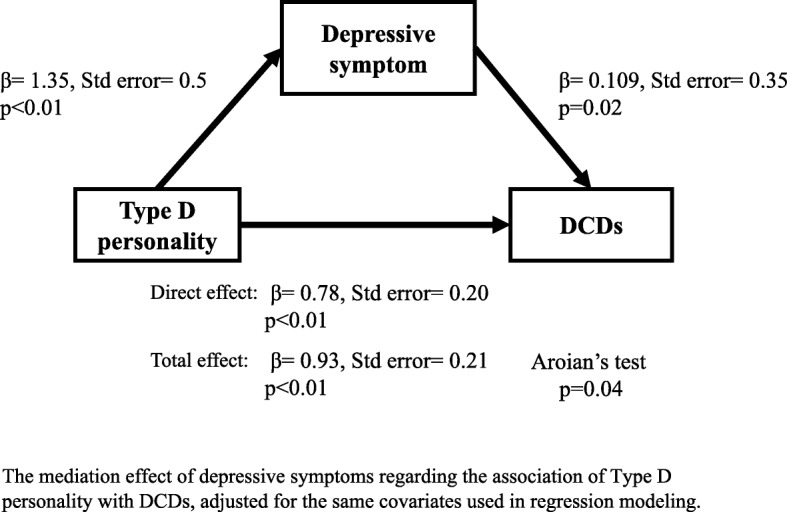
Mediation model for delirium/coma days. The mediation effect of depressive symptoms regarding the association of Type D personality with DCDs, adjusted for the same covariates used in regression modeling

The mediation analyses involved Type D personality (X; independent variable), depressive symptoms (M; mediator), and DCDs (Y; dependent variable) and were adjusted for the same covariate factors in regression modeling (Fig. 4). The analysis was performed according to Baron and Kenny’s method [42] as follows:

- ・First, Type D personality (X) significantly predicts DCDs (Y) (β = 0.93; *p* < 0.01).
- ・Second, Type D personality (X) significantly predicts depressive symptoms (M) (β = 1.35; *p* < 0.01).
- ・Third, in regression analysis, both Type D personality (X) and depressive symptoms (M) are predictors for DCDs (Y) (β = 0.78; *p* < 0.01), (β = 0.109; *p* = 0.02).
- ・The subsequent Aroian test, which tests the statistically significant difference in results between univariate and regression analyses with respect to Type D personality (X) for DCFDs (Y), was significantly different (*p* = 0.04).

Based on the above analysis, our present findings show that Type D personality is an independent predictor of DCDs and that depressive symptoms had a partial mediating effect on the relationship between Type D personality and DCDs after adjustment.

## Discussion

The present study is the first to demonstrate that Type D personality patients experience longer acute brain dysfunction (measured as delirium/coma days) during 7 days after operation, after adjusting for severity and various predicting factors. Although a previous study had shown that the prevalence of Type D personality is relatively high (46%) in Japan among healthy subjects [44], the present study is the first to show that the Japanese prevalence rates are comparable to European cardiac surgery patients [22]. One possible reason for the difference between the current findings and the previous Japanese study could be that the earlier study was conducted in the rural areas of Japan, which have a higher population of the elderly, thus inflating the prevalence of Type D personality.

Several previous studies showed that Type D personality was associated with depressive symptoms [21, 45] and these were in turn were associated with delirium [3, 46]. Our present results are in line with these earlier results but we differed in our methods by employing regression (including interaction) models and mediation modeling to analyze statistical significance within our findings that depressive symptoms have a partial mediating effect between Type D personality and acute brain dysfunction during the 7-day period after surgery.

Based on these analyses, we found a theoretical relationship between distressed personality and depressive symptoms [47]. Depressive symptoms can be said to have an additive deleterious effect on DCDs when combined with Type D personality. Thus, we should be aware that patients with Type D personalities may experience delirium and brain dysfunction after cardiac surgery and should be monitored carefully for depressive symptoms. Depressive symptoms are a solid predictive factor for delirium [48]; however, there is no knowledge of the association between Type D personality and depressive symptoms for prolonged acute brain dysfunction. We assume that Type D personality patients might underreport their symptoms even if they are in such an at-risk population for depression. Therefore, this propensity to underreport depressive symptoms underscores the need for solid evaluative tools to screen out Type D personalities from patient pools for more intensive monitoring to assist in their recoveries. We suggest further researches should focus on this interaction and mediation when studies for acute brain dysfunction include Type D personality or depressive symptoms as a factor. We also observed a NA and SI-independent effect for DCDs. From this result, we assumed that each component of the Type D personality worsens acute brain dysfunction after cardiovascular surgery. Previous research showed that SI modulates the effect of NA on cardiac prognosis following percutaneous coronary intervention [49]. Further research with a proper sample size is needed to check for any modulating effect for acute brain dysfunction.

Another potential mechanism through which Type D personality might have a negative influence on acute brain dysfunction may include inflammation and endothelial dysfunction. Previous observational studies showed that Type D personality was significantly associated with increased levels of IL-6 and TNF-α [50, 51]. In addition, another study showed that Type D personality is significantly associated with elevation of another pro-inflammatory marker, C-reactive protein [52], in a large, population-based study [45]. However, not only is Type D personality associated with inflammation, it is also linked to endothelial dysfunction. Interestingly, a previous study has reported that Type D personality is associated with decreased endothelial progenitor cells in patients with heart failure [53] and a recent study in patients with coronary artery disease showed that the association of Type D personality with endothelial dysfunction was robust across time [54]. It was already shown that inflammation biomarkers and these receptors associated with onset of delirium [55] and endothelial dysfunction associated with acute brain dysfunction during critical illness [56]. Further research is needed to explore whether the underlying mechanism of the observed relationship between Type D personality and delirium could be neural inflammation and/or endothelial factors.

### Limitation

There are several limitations in the present study. First, since this study is a cross-sectional design, the direction of the mediation between Type D personality and depressive symptoms cannot be confirmed. Second, the Type D personality scale (DS14) and depressive symptom scale (HADS) might have some overlapping questions. Additionally, the stress and dysphoria that naturally results from impending surgery might have skewed testing that was done the day before surgery. However, a previous study showed that Type D personality and depression are distinct manifestations of psychological distress [57]. Hence, we think that our current finding that shows a cross between independent variable and mediating effect might be valid. Third, despite the good response rate (90%), the non-consenting patients (who were not assessed) may have refused consent because of a higher level of depressive symptoms, leading to some bias in the results.

## Conclusion

Type D personality is a prognostic predictor for prolonged acute brain dysfunction (delirium/coma) in cardiovascular patients independent from depressive symptoms. Furthermore, Type D personality-associated depressive symptoms increase the magnitude of acute brain dysfunction.

## Abbreviations

CAM-ICU: Confusion Assessment Method for the ICU
CNS: Central nerve system
DCDs: Delirium/coma days
DS14: Type-D personality Scale-14
EuroSCORE II: European System score for Cardiac Operative Risk Evaluation II
HADS: Hospital Anxiety and Depression Scale
IL-6: Interleukin-6
MMSE: Mini-Mental State Examination
mSOFA: Modified Sequential Organ Failure Assessment
NA: Negative Affectivity
POLR: Proportional Odds Logistic Regression
RASS: Richmond Agitation - Sedation Scale
SD: Standard deviation
SI: Social Inhibition
TNF-α: Tumor Necrosis Factor α
VIF: The variance inflation factor
